# A program quality framework: a collaborative teaching team approach to quality assurance, quality enhancement and staff capacity building

**DOI:** 10.3389/fmed.2023.1242408

**Published:** 2023-08-31

**Authors:** Thea van de Mortel, Creina Mitchell, Mary-Ann Shuker, Judith Needham, Victoria Kain, Georgina Sanger, Beth Pierce

**Affiliations:** ^1^School of Nursing and Midwifery, Griffith University, Southport, QLD, Australia; ^2^Griffith Health, Griffith University, Southport, QLD, Australia; ^3^School of Nursing and Midwifery, Griffith University, Meadowbrook, QLD, Australia; ^4^School of Nursing and Midwifery, Griffith University, Nathan, QLD, Australia

**Keywords:** curriculum enactment, quality assurance, quality enhancement, framework, faculty professional development

## Abstract

A global shortage of registered nurses provides a further impetus to retain nursing students and graduate safe nurses. While various frameworks support curriculum design and describe the need for ongoing curriculum evaluation and monitoring, there is little in the literature to support the enactment and ongoing quality enhancement of curricula. Translation of the curriculum plan into the delivered curriculum relies on academics who may or may not be adequately prepared for course writing and teaching in higher education settings, despite their discipline expertise. Additionally, there are well recognized issues of curriculum drift where curriculum innovations and changes are whittled away over time by incremental changes to courses that interfere with the integrity of the accredited curriculum. We propose an evidence-based Program Quality (ProQual) Framework that takes a holistic, collaborative, and systematic approach to monitoring and enhancing curriculum quality and program delivery over the life of the curriculum while developing staff capability and scholarship.

## 1. Introduction

Baccalaureate-prepared registered nurses (RNs) decrease patient mortality (1). However, the current global shortage of RNs is projected to worsen (2). Thus, there is an ongoing impetus to provide high-quality nursing curricula to ensure safe graduates and a positive student experience to retain nursing students until graduation. While definitions differ, quality can be defined as a level of excellence that can be assured–through reaching benchmarks–and enhanced over time (3).

Whilst referring to higher education at the institutional level, Land and Gordon (4) indicate that higher education institutions are entrusted with public money to develop human capital and suggest that frameworks that strengthen core academic processes are the best way to protect these interests. In this paper we propose an evidence-based framework to protect and enhance curriculum quality and program delivery through a capacity-building and collaborative team approach. Throughout this paper, ‘program’ refers to a degree program, and ‘course’ to subject level units within the program.

Nursing programs undergo accreditation as one means of ensuring they provide high-quality education (5). Australian accreditation standards mandate:

1. stakeholder consultation to inform curriculum development,
2. an underpinning curriculum framework,
3. mapping that demonstrates constructive alignment between course and program learning outcomes, the syllabus and assessment,
4. the minimum clinical hours to be completed,
5. that national RN and health standards are addressed,
6. that there are student support mechanisms and a documented process to ensure appropriate teacher qualifications, and
7. that governance mechanisms include quality assurance processes that seek and respond to stakeholder feedback and inform the accrediting body of curriculum changes (6).

Once the proposed curriculum (in what is effectively an outline form) is accredited, it is handed to the teaching staff to enact. The enactment of a quality curriculum is predicated on various assumptions. The first is that having postgraduate qualifications and clinical expertise translates to skill in course development and teaching. However, these do not automatically qualify someone as either a competent course writer or teacher (7); clinicians moving into academia often lack formal teaching and course convening preparation and feel unprepared for the role (8).

Another assumption is that teaching staff are eager to adopt innovations. Historically, modifications to courses have often been made using a ‘course’ rather than ‘program’-based focus, and staff tend to return their courses via incremental tweaks to what they are comfortable teaching, with ensuing curriculum drift (9). Decisions about andragogy can be made based on prior experience rather than on solid evidence (5). The enacted curriculum is then not the planned curriculum described in the accreditation documents (10), and ‘eventually resembles its pre-innovative ancestor’ [(11), p. 2]. Additionally, in large multi-campus programs, maintaining consistency in course delivery across multiple staff and campuses remains a challenge.

Ralph and colleagues’ (5) curriculum design framework recommends an evidence-based best practice approach is taken to curricula and that processes are put in place to review and monitor quality. Little is written about mechanisms to do this through the life of a curriculum. Quality in curriculum translation, implementation, and evaluation, as well as nursing faculty development is frequently addressed piecemeal. The literature on quality initiatives is often focused on improving the teaching of particular content, e.g., health policy (12).

## 2. Discussion

**Figure 1:**
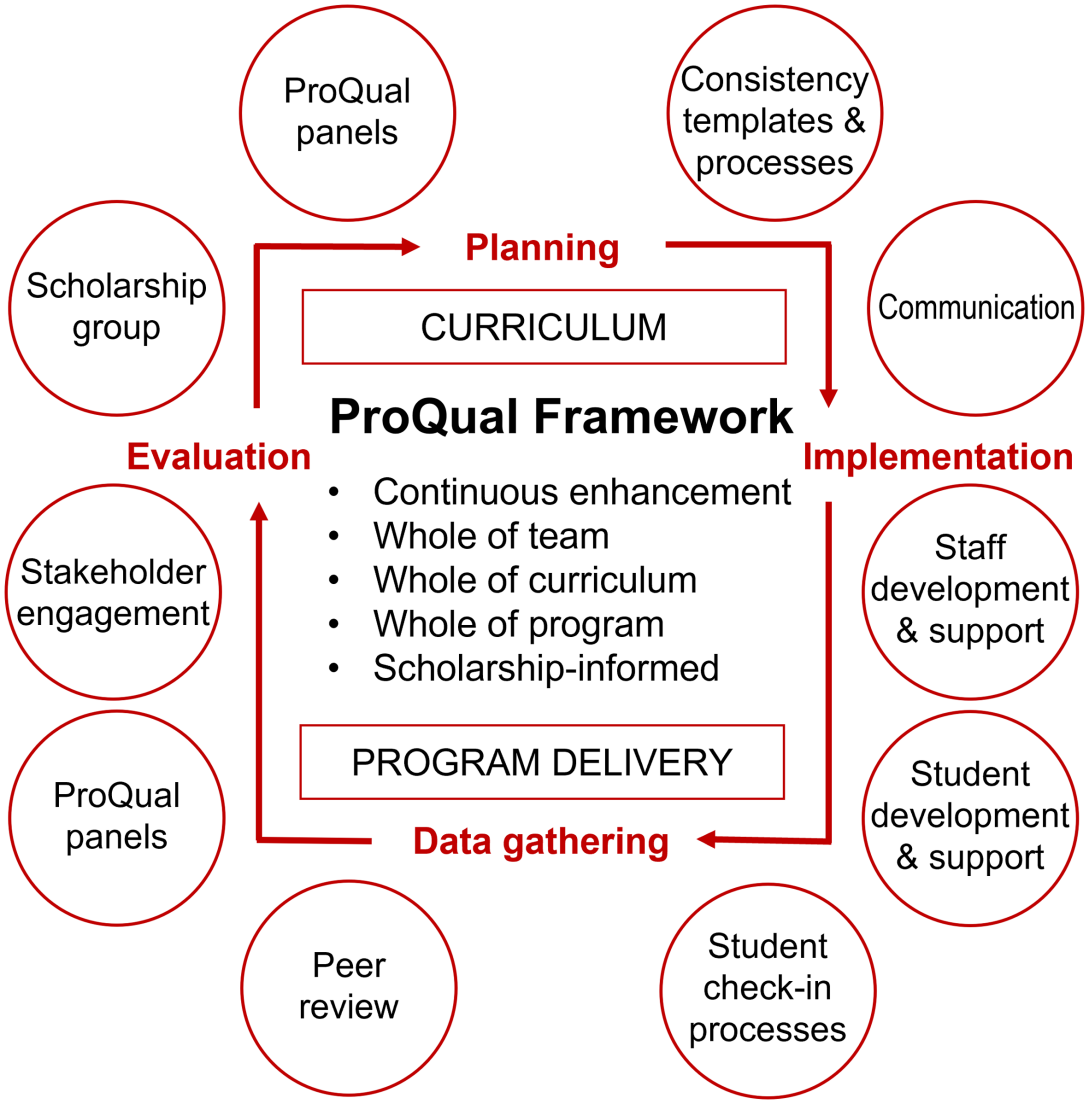
ProQual framework. Adapted from Tague (13).

To address these issues, we propose an overarching evidence-based Program Quality (ProQual) Framework that takes a holistic, collaborative and systematic approach to monitor and enhance the quality of the curriculum and program delivery while developing staff learning and teaching capability and scholarship (Figure 1).

The ProQual Framework’s planning, implementation, data gathering, and evaluation phases incorporate elements of existing quality assurance approaches such as the Plan-Do-Study-Act cycle, which was developed for quality assurance in business (13). The ProQual Framework focuses on the translation, delivery, and evaluation phases of the curriculum, and:

1. involves the whole teaching team plus a curriculum lead, educational designers, Program Director, and Deputy Head of School (Learning & Teaching),
2. is conducted on a trimester-by-trimester basis throughout the life of the curriculum (continuously and iteratively),
3. incorporates both quality assurance and quality enhancement mechanisms,
4. is underpinned by the scholarship of learning and teaching,
5. is informed by regular stakeholder feedback,
6. capacity-builds staff and students, and
7. takes a ‘whole of program’ approach to curriculum modifications to minimize curriculum drift and ensure that constructive alignment and scaffolding - and thus curriculum integrity-is maintained throughout the life of the curriculum.

### 2.1. ProQual panels

Kelder et al. (14) indicate the importance of engaging teachers in collaborative ongoing curriculum evaluation. The continuous curriculum review process developed and evaluated by van de Mortel and Bird (9), van de Mortel et al. (15), and Bird et al. (16) provides an effective mechanism to do this. While departments ordinarily have a committee to review planned changes to courses/programs, these often operate at the department rather than program level, have limited attendance, and due to the volume of business to be debated and approved, do not generally enable deep consideration of how proposed changes might impact program scaffolding, program leaning outcomes and constructive alignment.

A trimester-by-trimester ProQual panel addresses these challenges, focusing on collaborative social learning in the manner of a community of practice (17) to:

1. facilitate peer-to-peer learning that enhances members’ capabilities,
2. minimize curriculum drift, and
3. enhance course/program decision-making through a whole of program/whole of team approach.

Prior to the start of trimester, the ProQual panel meets to plan upcoming program delivery. Planned changes from previous course iterations are discussed to ensure they will be completed prior to delivery to close the loop. A program level assessment matrix ensures that assessments are spaced to reduce pressure on students (9). The panel also meets after course delivery to discuss course and program challenges and successes, facilitating whole team deliberations on any proposed changes. The ProQual panel is underpinned by Bandura’s (18) Social Cognitive Theory; the collaborative discussion of proposed course changes against concepts of constructive alignment and scaffolding, facilitates both direct learning about aspects of quality curricula, and vicarious learning-through discussion of other academics’ experiences and ideas. It further establishes staff ownership and understanding of the curriculum.

### 2.2. Consistency templates and peer review

As well-organized learning materials positively impact student engagement and higher-order learning (19), consistency templates, and peer review – a fundamental component of the dissemination of knowledge (20) and academic development (21) – are used to assure the quality of course resources. Course content and sites are developed using a template that assures a professional, consistent, easy-to-navigate structure, and embeds sound andragogical approaches to encourage active learning, which improves students’ learning outcomes (22). Peer, curriculum lead and educational designer reviews are conducted prior to the release of course sites to ensure quality. Secondly, a consistency template is used for assessment task descriptions to ensure that assessments contain key information in a logical standardized format. Peer and educational designers also review assessment task descriptions and rubrics prior to approval by the Program Director for release to students. This ensures the task description is clear, learning outcomes are addressed, and the rubric and task description are aligned as part of pre-marking moderation. Peri-and post-marking moderation are also conducted.

### 2.3. Communication

With large, multi-campus programs it is critical to provide clear communication and consistent decision-making across campuses to ensure equity for students. Our program leadership team meets weekly to discuss implementation issues and problem solve. Teaching staff are kept informed through a weekly electronic newsletter and quarterly ProQual panels. To ensure consistent decision-making in courses with work-integrated learning (WIL), a Clinical Reference Group meets bi-annually, providing a mechanism to obtain feedback from clinical teams on issues related to students’ WIL placements, and to communicate updates in processes. Teaching staff and students are also supported with WIL placement guidelines that explain placement processes. Each course has a primary convenor responsible for overall communication with students and a communication plan that details key messages to guide student learning and encourage engagement, while minimizing *ad hoc* messaging.

### 2.4. Staff support, development and scholarship

Sidhu (7) indicates higher education institutions have a responsibility to ensure educators are competent. ProQual panels provide professional development through workshops on topical issues. The iterative discussion of proposed curriculum changes and issues related to program delivery provide opportunities for staff to view examples of best practice or solutions to issues. The peer review process also provides formative learning opportunities (23).

Learning and teaching scholarship should underpin course design and teaching practice (5, 15). Teaching faculty are encouraged to join a Scholarship of Effective Learning and Teaching (SELT) community of practice dedicated to:

1. improving student learning, the student experience, program retention and graduate success,
2. driving innovation, and
3. supporting excellence in teaching, recognition of exemplary practice, and building leadership.
  Mentoring is provided through peer review of draft manuscripts, abstracts and grant applications and brainstorming research ideas during SELT meetings. Staff recognition for educational practice is supported through advice on, and peer review of, teaching award and Higher Education Academy Fellowship applications. Group meetings provide opportunities for networking and for staff to join scholarship teams in an area of interest, creating a culture of evidence-based teaching practice. Staff are encouraged to plan evaluations of innovations to inform their teaching and provide evidence of outcomes for teaching award applications. Academics are supported to complete postgraduate studies in learning and teaching and funding can be sought for professional development.

### 2.5. Student development and support

Lizzio (24) describes the Five Senses of Success that positively impact retention and student success: students’ sense of purpose, connection, capability, resourcefulness, and academic culture. These underpin our student lifecycle program. Commencing students are offered a two-day orientation (25) that incorporates connectedness activities and builds sense of purpose. A weekly Nurses Connect electronic student newsletter provides just in time information for students, advertises development opportunities and reduces *ad hoc* email traffic. Regular interaction promotes connectedness, and, as a result, retention (24). We also conduct extra-curricular weekly academic skills development workshops for commencing students and Peer Assisted Study Sessions for our bioscience courses to build capability and resourcefulness. We have a Student Lifecycle team dedicated to student support throughout the student lifecycle. Year level coordinators coordinate the academic skills programs, implement employability initiatives, and provide pastoral care (26). A peer mentoring program provides further support and connectedness opportunities. Students are thus supported with both transition in (to university), through, and out (to graduate positions) (25).

### 2.6. Student check-in and stakeholder engagement

An early in-house anonymous survey with students in each course is used to get a sense of the student experience of the course to detect and address any issues (26). This complements university level surveys of commencing students to determine their experiences of orientation and the early teaching weeks, which are based on Lizzio’s (24) Five Senses of Success. The Lifecycle team develops an annual student lifecycle plan, assesses student feedback, and implements strategies to improve the student experience. We also respond to end of course and WIL feedback. Regular industry stakeholder feedback on our programs and graduates is sought through external advisory committee meetings.

### 2.7. Outcomes

We implemented the ProQual framework in 2016 to drive quality enhancement of our new curriculum. While aspects of this have been previously positively evaluated (9, 15, 16, 27, 28), we have not formally evaluated the full framework, however, over the life of the 2016–2021 curriculum, courses with satisfactory student ratings increased by 15%, program retention by 7% and admissions rank cut-offs (an indicator of student demand/program reputation) by 17–21 points. Three years after implementing the SELT group, scholarly publications and conference presentations had doubled.

### 2.8. Limitations

This framework was developed for a nursing program and thus may not be fully generalisable to other discipline areas, for example, those that do not include WIL. It may also be more difficult to implement in programs where many courses are shared between different degree programs due to the level of consultation required. However, the underlying principles - which focus on consistency, peer review, communication, as well as staff and student development and support - can be used by any degree program to ensure curriculum integrity, develop staff and enhance student satisfaction and retention. While the proactive processes that form the framework have a definite time cost, we have found this is offset to some extent by a reduction in foreseeable problems, a reduced need for reactive management of issues, and a substantial reduction in student appeals of grades (Box 1), and the other benefits noted above make it worth the effort.

BOX 1 **Practice example**

In response to multiple Review of Decision of Grade requests from students in 2015, the ProQual panel examined the issues in relation to student appeals of grades. One of the issues identified was that some task descriptions lacked clarity or marking criteria did not fully align with the task description – confusing students (and at times markers) and providing grounds for appeal.

Consequently, an assessment template was created by the Program Director – with feedback from the team – to improve the clarity of assessment tasks and marking criteria. The template was coupled with robust peer review of assessments by a curriculum consultant and the Program Director, which complemented existing marking moderation processes. Requests for Reviews of Decision of Grade declined by 75% following implementation of these processes.

## 3. Conclusion

We suggest the ProQual Framework shifts the focus from sub-optimal individual course-based practices to a collaborative programmatic scholarship-based approach to maintain curriculum integrity, assure and enhance curriculum quality and program delivery, and develop academic staff into the future.

## Data availability statement

The original contributions presented in the study are included in the article/supplementary material, further inquiries can be directed to the corresponding author.

## Author contributions

TM led framework conceptualization with critical input from BP, CM, M-AS, JN, GS, and VK. TM drafted the manuscript. All authors made revisions, approved the final version for publication and agreed to be accountable for all aspects of the work.

## Conflict of interest

The authors declare that the research was conducted in the absence of any commercial or financial relationships that could be construed as a potential conflict of interest.

## Publisher’s note

All claims expressed in this article are solely those of the authors and do not necessarily represent those of their affiliated organizations, or those of the publisher, the editors and the reviewers. Any product that may be evaluated in this article, or claim that may be made by its manufacturer, is not guaranteed or endorsed by the publisher.
